# The role of GPD1L, a sodium channel interacting gene, in the pathogenesis of Brugada Syndrome

**DOI:** 10.3389/fmed.2023.1159586

**Published:** 2024-06-19

**Authors:** Alexander M. Greiner, Haider Mehdi, Chloe Cevan, Rebecca Gutmann, Barry London

**Affiliations:** ^1^Division of Cardiovascular Medicine, Department of Internal Medicine, University of Iowa Carver College of Medicine, Iowa City, IA, United States; ^2^Department of Internal Medicine, Abboud Cardiovascular Research Center, University of Iowa Carver College of Medicine, Iowa City, IA, United States; ^3^University of Iowa Interdisciplinary Graduate Program in Genetics, Interdisciplinary Graduate Program in Genetics, University of Iowa, Iowa City, IA, United States

**Keywords:** inherited arrhythmia syndrome, Brugada Syndrome (BrS), sudden cardiac death (SCD), exome sequencing, genetics, arrhythmia

## Abstract

**Background:**

Brugada Syndrome (BrS) is an inherited arrhythmia syndrome in which mutations in the cardiac sodium channel *SCN5A* (Na_V_1.5) account for approximately 20% of cases. Mutations in sodium channel-modifying genes may account for additional BrS cases, though BrS may be polygenic given common SNPs associated with BrS have been identified. Recent analysis, however, has suggested that *SCN5A* should be regarded as the sole monogenic cause of BrS.

**Objective:**

We sought to re-assess the genetic underpinnings of BrS in a large mutligenerational family with a putative mutation in *GPD1L* that affects surface membrane expression of Na_V_1.5 *in vitro*.

**Methods:**

Fine linkage mapping was performed in the family using the Illumina Global Screening Array. Whole exome sequencing of the proband was performed to identify rare variants and mutations, and Sanger sequencing was used to assay previously-reported risk single nucleotide polymorphsims (SNPs) for BrS.

**Results:**

Linkage analysis decreased the size of the previously-reported microsatellite linkage region to approximately 3 Mb. GPD1L-A280V was the only coding non-synonymous variation present at less than 1% allele frequency in the proband within the linkage region. No rare non-synonymous variants were present outside the linkage area in affected individuals in genes associated with BrS. Risk SNPs known to predispose to BrS were overrepresented in affected members of the family.

**Conclusion:**

Together, our data suggest GPD1L-A280V remains the most likely cause of BrS in this large multigenerational family. While care should be taken in interpreting variant pathogenicity given the genetic uncertainty of BrS, our data support inclusion of other putative BrS genes in clinical genetic panels.

## 1 Introduction

Brugada Syndrome (BrS) is an inherited arrhythmia syndrome characterized by ST-segment elevation in the right precordial leads of the electrocardiogram (EKG; V_1_ through V_3_) and sudden cardiac death (1). The molecular mechanism underpinning BrS was initially described as autosomal-dominant loss-of-function mutations in the main cardiac sodium channel, Na_V_1.5, encoded on chromosome 3 by *SCN5A* (2). These loss-of-function mutations decrease inward depolarizing sodium current, which can result in premature repolarization of the epicardium in the right ventricle, slowed conduction, ventricular tachyarrhythmias, and sudden cardiac death (3). However, only approximately 20% of patients with Brugada Syndrome have mutations in *SCN5A*. The advent of massively parallel sequencing has allowed many groups to investigate the genetic underpinnings of BrS over the past two decades. These genetic data, in combination with molecular studies, have expanded the number of putative disease-causing genes in BrS from one gene, *SCN5A*, to over 20 genes today. Genes reported to have mutations which cause BrS can be broadly classified as those which encode (1) sodium channels, (2) sodium channel interacting proteins, (3) other ion channels, and (4) metabolic proteins (4). Recent reports have also suggested BrS may be polygenic in nature, and that single nucleotide polymorphisms (SNPs) near SCN5A-SCN10A and loci responsible for *SCN5A* transcription and trafficking are associated with BrS (5, 6).

Our laboratory used mapping with microsatellites in a large, multigenerational family with BrS to identify a linkage region on chromosome 3 with a LOD score >4 that did not contain *SCN5A* or *SCN10A* (7, 8). Positional cloning and subsequent molecular analysis identified a variant in glycerol 3-phosphate dehydrogenase 1-like (GPD1L) as the putative cause of this family's disease. Peak sodium current in HEK293 cells transfected with Na_V_1.5 decreased if cells were co-transfected with GPD1L-A280V compared to those co-transfected with GPD1L-WT. Additional studies suggested GPD1L-A280V decreases Na_V_1.5 membrane expression, and that GPD1L mutations were associated with sudden infant death syndrome (SIDS) on molecular autopsies (9). The identification of GPD1L by linkage and the subsequent demonstration that it decreases sodium channel membrane localization and current together suggests GPD1L-A280V is pathogenic for BrS.

The explosion of genetic data, especially variants of uncertain significance, from clinical genetic testing has led to a plethora of putative mutations but a dearth of scientific evidence supporting the pathogenicity of these variants. Concerns about variants of uncertain significance being classified as pathogenic without data to support the claim has been reviewed in many publications (10, 11). This concern sparked a review of the pathogenicity of reported BrS genes using the Clinical Genome Resources (ClinGen) framework. This review determined that *SCN5A* was the only gene with sufficient scientific evidence to definitively be regarded as a causative gene for BrS (12). *GPD1L* was excluded due to a large linkage region in which other genes had not been sequenced and the relatively high allele frequency of GPD1L-A280V (gnomAD V2.1.1 allele frequency = 1.29E-4) (13, 14).

In the present study, we provide high-depth whole exome sequencing data in the proband of our originally-reported large multigenerational family, SNP-based linkage analysis of affected individuals, and sequencing data of previously defined BrS risk SNPs to support our initial reports that the A280V mutation in GPD1L, a sodium channel interacting protein, is pathogenic for BrS.

## 2 Methods

### 2.1 Patient enrollment and phenotype validation

Patients were enrolled under protocols approved through the University of Pittsburgh and University of Iowa Institutional Review Boards (IRBs). All individuals in the study provided informed consent for participation in the study. Type 1, Type 2, or Type 3 Brugada Syndrome, or clinically unaffected status, was determined through clinical history, electrocardiogram analysis, and clinical provocative testing using procainamide as previously described (8).

### 2.2 Genomic DNA isolation and RNAse A treatment

Genomic DNA was isolated from peripheral blood using commercially-available kits or automated machine as previously described (8). Isolated DNA was treated with RNAse A and re-isolated by ethanol precipitation prior to sequencing. DNA integrity was assessed by agarose gel electrophoresis and quantified using Qubit High Sensitivty DNA assays (ThermoFisher Scientific).

### 2.3 SNP calling and linkage analysis

Ten affected and five unaffected individuals from the family had genomic DNA prepared as described above. After passing quality control and subsequent library preparation, SNPs were called using the Infinium Global Screening Array (Illumina) at CD Genomics (Shirley, NY, USA) which sequences approximately 654,027 SNPs. Call rates were greater than 98% for all individuals (Supplementary Table 1). Chromosomes were phased using ShapeIt2 with DuoHMM enabled and the reference HapMap Phase II data (15, 16). The resultant phased haplotypes were investigated manually for recombination using our previously-reported microsatellite data as the starting point. Logarithm of odds scores were calculated using Superlink-Online SNP (17).

### 2.4 High-depth whole exome sequencing and analysis

Whole exome sequencing was performed at the Iowa Institute of Human Genetics (IIHG) on genomic DNA prepared as described above. Library preparation was performed using standard protocols for the Agilent SureSelect V6 + UTR library preparation kit (Agilent Technologies). The resulting 150 base pair paired-end library was sequenced on an Illumina HiSeq 4000. Resultant .fastq files underwent quality control using FastQC (18). Alignment, variant calling, and variant annotation were performed using BWA-MEM (alignment), GATK4 (variant calling), and SnpEff (variant annotation) using an implementation of BCBIO and the GRCh37 reference genome (19–24). A second variant calling tool, Freebayes, was used in a near-identical pipeline to ensure consensus variant calls were achieved (25). Greater than 80% of targeted bases had greater than 50x coverage (Supplementary Figure 1). A brief summary of sequencing results is shown in Supplementary Table 2.

Two approaches were used to identify candidate variants (1) a linkage-region focused analyses and (2) a linkage-naive approach. The linkage-region focused analysis was limited to the newly-defined linkage region on chromosome 3, while the linkage-naive approach entertained variants on any autosome. For both approaches, the resulting annotated variant call file was a used to generate a database compatible with Gemini (26). Variants present at less than 1% minor allele frequency, a lenient threshold for autosomal dominant disease, were included. Variants were then segregated according to impact (nonsense, splicing, missense, synonymous) with synonymous variants removed from consideration. Variant location information, either exonic, UTR, intronic, or intergenic, was subsequently used in prioritization of variants.

### 2.5 Sanger Sequencing of Risk SNPs

Sanger sequencing of BrS risk SNPs in *SCN5A, SCN10A*, and *HEY2* was performed at the Iowa Institute of Human Genetics using custom primers designed in-lab and synthesized by Integrated DNA Technologies (Supplementary Table 3). The resulting chromatograms were analyzed using NCBI Blast, Finch, and SnapGene from which wild-type, heterozygous, or homozygous SNP calls were made and manually validated.

### 2.6 Protein and variant modeling

A single unpublished protein model of GPD1L is present on the RCSB Protein Databank (26, 27). AlphaFold computational models for GPD1L are also publicly available from EMBL. The AlphaFold GPD1L model AF-Q8N335-F1-model_v1 was downloaded and imported into PyMol (28). The mutagenesis function of PyMol was used to visualize the impact of the three predicted GPD1L-A280V rotamers. Strain scores were calculated for all rotamers within PyMol (Supplementary Figure 2). Variant data for *GPD1L* was downloaded from ClinVar (29).

## 3 Results

### 3.1 Linkage analysis defines the region on chromosome 3 that co-segregates with Brugada Syndrome

**Figure 1:**
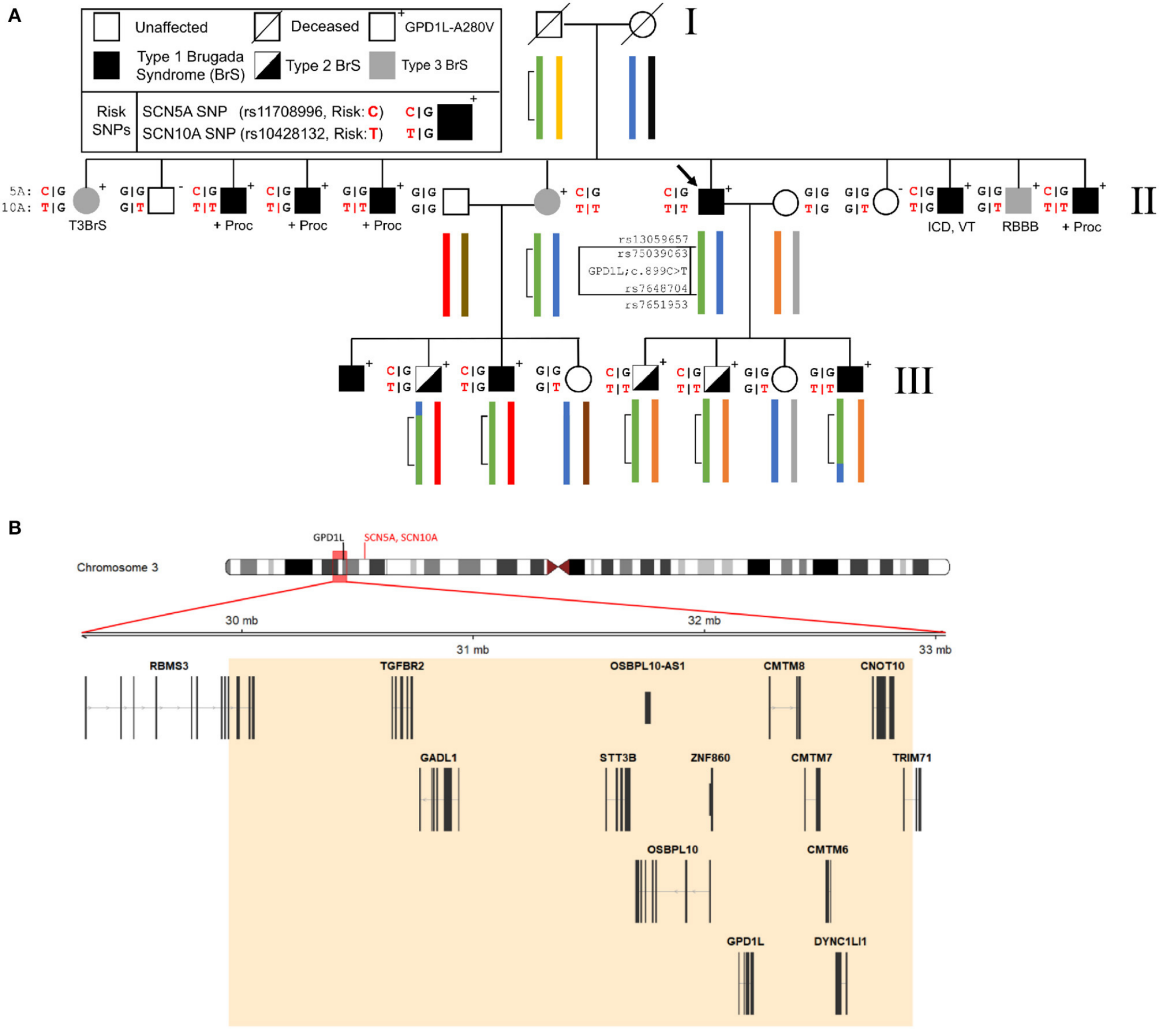
Representative pedigree of the family, linkage analysis, and assessment of Brugada Syndrome (BrS) risk SNPs. **(A)** Linkage analysis demonstrates a small region on chromosome 3 (inset map and green bracketed region) which has undergone recombination in the third generation of the pedigree. Previously reported risk SNPs (SCN5A and SCN10A, red) for the BrS phenotype are associated with the GPD1L-A280V. **(B)** The 3 Mb region on chromosome 3 identified by linkage analysis contains 14 genes, including *GPD1L*. *SCN5A* and *SCN10A* lie upstream of GPD1L (ideogram, red text) and outside of the refined linkage region (ideogram, red box). ICD, implantable cardioverter defibrillator; +Proc, positive procainamide challenge; RBBB, right bundle branch block; VT, ventricular tachycardia.

To more precisely define the linked genomic regions within this family we performed linkage analysis using the Illumina Global Screening Array with ten affected and five unaffected family members. The previously-identified 15 cm linkage region in this family which we reported was used as a starting point for our investigation (8). Analysis of phased haplotypes identified a narrowed linkage region defined by the SNPs rs13059657 and rs7651953 (GRCh37, chr3:29,899,567-32,970,737) (Figure 1). Individuals III-2 and III-8 defined the 5′ and 3′ breakpoints, respectively. Calculation of affected only LOD scores using individuals having a Type 1 EKG pattern or positive procainamide test using Superlink-Online SNP results in a LOD score of 2.02 for this region (17). The addition of individuals with Type II EKG patterns results in a LOD score of 3.18. The region of interest contains 14 genes, including *GPD1L*, but not *SCN5A* or *SCN10A* which are approximately 6 Mb downstream (Figure 1).

### 3.2 Whole-exome sequencing analysis identifies GPD1L-A280V as the only rare exonic variant in the linked region

High-depth (100x) whole exome sequencing of the proband was performed using the Agilent SureSelect V6+UTR capture kit. Limiting the analysis to the newly defined linkage region identified GPD1L-A280V as the only rare, exonic coding variation. No other exonic or splice variants occurred at an allele frequency of less than 1% in or near this region.

Outside of the linkage region, the proband carried no variants that were annotated as pathogenic for cardiovascular diseases in the ClinVar database. In addition, he carried no rare missense or nonsense variants in genes previously assocated with BrS or that have known interactions with Na_V_1.5. He was heterozygous for a synonymous SNP in CACNA1C (p.G1738G;c.5358C>T, *f* = 1.21E-5), and for non-synonymous missense variants of uncertain significance in genes associated with cardiomyopathy and arrhythmia, including a variant in ANK2 (p.R2416G;c.7246C>G, unreported), a variant in FHL2 (p.V187M;c.559G>A, *f* = 6.78E-5), and two variants in TTN (p.A31503T;c94507G>A, *f* = 5.04E-5 and p.F14410C;c.43229T>G, unreported) (Table 1). Linkage analysis and Sagner sequencing confirmed that none of these variants cosegregated with BrS in this family.

**Table 1:** Variants in reported Brugada Syndrome Genes or in the Inherited Cardiac Condition (ICC) Gene List.

**Gene list**	**Position**	**Consequence**	**Gene**	**Frequency**
Linkage region	chr3:32200588C>T	p.Ala280Val	GPD1L	1.29E-04
BrS	chr12:2788732C>T	p.Gly1738Gly	CACNA1C	1.21E-05
ICC	chr2:105979871C>T	p.Val187Met	FHL2	6.78E-05
	chr2:179411745C>T	p.Ala31503Thr	TTN	5.04E-05
	chr2:179497504A>C	p.Phe14410Cys	TTN	Unreported
	chr4:114277020C>G	p.Arg2416Gly	ANK2	Unreported
	chr16:15844004G>A	p.His690His	MYH11	5.25E-04

### 3.3 The SCN5A and SCN10A BrS risk SNPs are linked to GPD1L-A280V in this family

**Figure 2:**
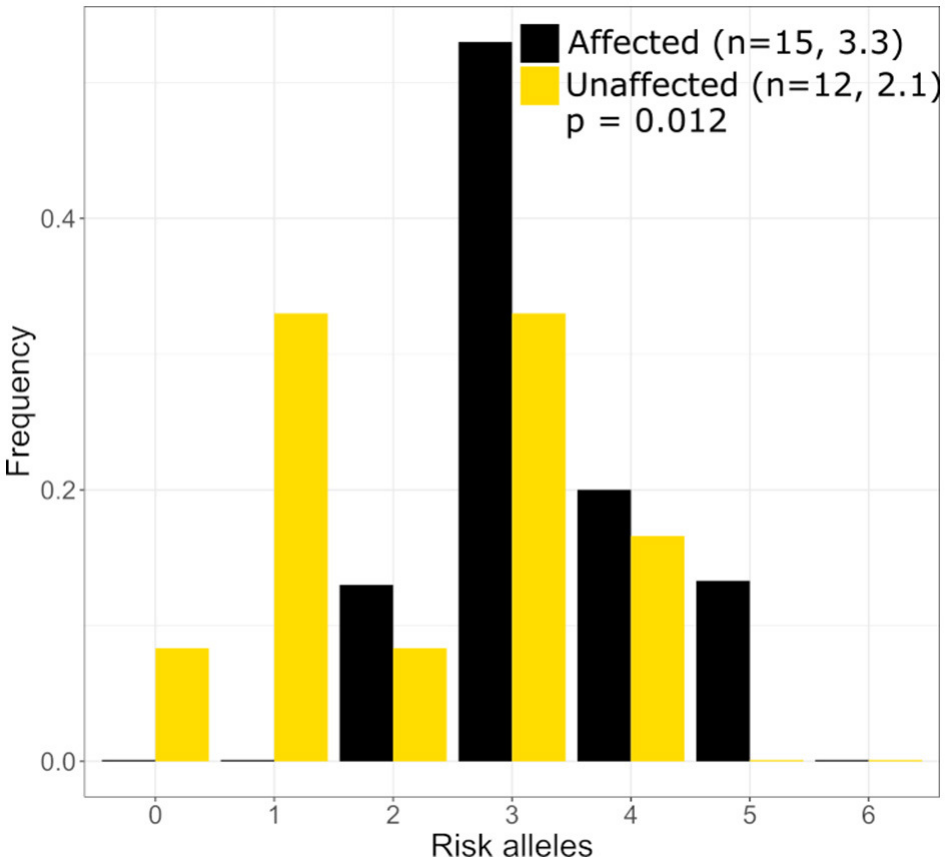
Distribution of Brugada Syndrome Risk Polymoprhisms in the large mutli-generational family. Individuals with Brguada Syndrome in our family carry more risk polymorphisms on average (3.3) than unaffected individuals (2.1) in our large mutli-generational family. The distribution of risk polymorphisms is similar to that which was reported by Bezzina et al. (5).

Three published risk SNPs for BrS [rs10428132 (*SCN10A*; total minor allele frequency, dbSNP (MAF) = 0.40), rs11708996 (*SCN5A*; MAF = 0.14), and rs9388451 (*HEY2*; MAF = 0.42)] identified by Bezzina et al. were assessed in family members with sufficient DNA, showing 8/15 affected subjects (carrying the *GPD1L* putative mutation) in generations II and III are homozygous for the SCN10A risk allele and 12/15 subjects are heterozygous for the SCN5A risk allele (Figure 1). SCN5A and SCN10A are near GPD1L on chromosome 3; in most affected individuals the GPD1L-A280V putative mutation co-segregated with one SCN5A and one SCN10A risk allele. The HEY2 risk SNP on chromosome 6 segregated in Mendelian fashion. Overall, individuals affected with BrS (*n* = 15, including one individual not shown in Figure 1) carried 3.3 ± 0.2 risk SNPs, while unaffected individuals (*n* = 12) carried 2.1 ± 0.4 risk SNPs (*p* = 0.012) (Figure 2). The SCN5A and SCN10A risk SNPs carried along with GPD1L-A280V in this family may potentiate the pathogenicity of the GPD1L-A280V variant.

### 3.4 GPD1L-A280V may introduce protein instability in computationally-predicted models

**Figure 3:**
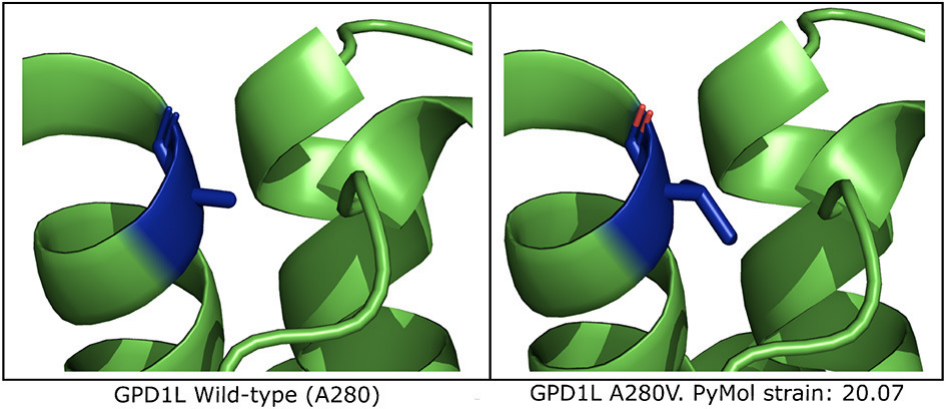
Computational modeling of GPD1L suggests A280V introduces steric clashing. **(Left)** The wild-type GPD1L structure as calculated by AlphaFold (AF-Q8N335-F1-model_v1) with residue A280 highlighted in blue. A280 resides in an alpha helix. **(Right)** One of three putative GPD1L-A280V rotamers as calculated by PyMol. All three predicted rotamers introduce strain in the alpha helix.

*In silico* mutagenesis of the A280 residue to V280 in a computational model of GPD1L demonstrates increased strain within the alpha helix in which residue 280 resides (Figure 3 and Supplementary Figure 2). Thus, *in silico* mutagenesis results in increased computationally-predicted strain stores indicating GPD1L-A280V may introduce steric hindrance in the GPD1L tertiary structure.

## 4 Discussion

### 4.1 Evidence supporting GPD1L as a causal gene for BrS and sudden death

The report by Hosseini et al. (12) using the Clinical Genome Resources (ClinGen) framework downgraded GPD1L as a potential cause of BrS, due to (1) the initial large reported linkage region (>14 MB); (2) the failure to fully screen all of the genes in the region for mutation, which was limited by the technology available in 2007; and (3) the relatively high allele frequency of GPD1L-A280V. Our analysis of this large, multigenerational family narrows the linkage region to approximately 3.1 Mb which includes *GPD1L* but excludes *SCN5A* and *SCN10A*. Whole exome sequencing confirmed GPD1L-A280V as the only rare exonic variant at an allele frequency < 1% within this region. The affected-only LOD score of 3.18 for individuals displaying Type I and Type II BrS patterns (Type 1 BrS alone = 2.02) is consistent with the previous reported LOD score of >3 which included unaffected individuals. In addition, while the A280V variant is relatively common in white people and south Asian people (minor allele frequency 1/6,000), this does not preclude pathogenicity in a founder population or fully account for incomplete penetrance (30).

Our findings show that BrS associated with *GPD1L* mutations behaves similarly to BrS caused by *SCN5A* and other unidentified mutations. The Type III BrS EKG pattern observed in patient II-7, an obligate carrier whose male children display Type I and Type II EKG patterns, exemplifies the incomplete penetrance more common in women and reported in other BrS patients (13, 31) (Figure 1).

**Figure 4:**
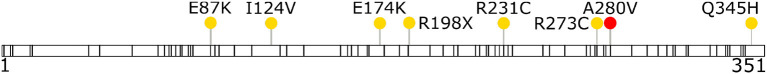
Reported GPD1L variants in ClinVar show GPD1L-A280V is not the sole reported putative mutation. Missense variants with conflicting interpretation (yellow circles, with GPD1L-A280V highlighted as red) and variants of uncertain significance (black bars, 83 total) were downloaded from ClinVar (accessed November 12, 2021) and plotted along the full length of GPD1L. All five variants with conflicting interpretation, including GPD1L-A280V, were submitted to ClinVar by their respective submitters with Brugada Syndrome as the clinical condition.

GPD1L-A280V is not the lone reported *GPD1L* putative mutation (Figure 4). Three putative mutations, p.E83K, p.I124V, and p.R273C), were identified in infants by molecular autopsy of 83 cases of sudden unexplained death and 221 cases of sudden infant death syndrome (9). As was found for A280V, each of these GPD1L putative mutations decreased peak sodium current in heterologous expression system or neonatal cardiac myocytes. A loss-of-function GPD1L mutation (p.R189X; c.565C>T) was reported to cosegregate in a small family with ventricular tachycardia and sudden death (32). The EKG of the proband showed a Type 2 BrS pattern [Huang et al. (32), Figure 1B]. In addition, ClinVar reports 3 other variants of uncertain significance in GPD1L (p.E174K, p.R231C, Q345H) (33).

### 4.2 The molecular and genetic basis of BrS

Mutations in *SCN5A* that disrupt Na_V_1.5 expression, trafficking, or function and decrease peak depolarizing sodium current are the predominant genetically-identifiable cause of BrS (1). The decrease in inward current is postulated to either cause premature repolarization or result in marked conduction slowing and block in the right ventricular outflow tract and septum, where the repolarizing transient outward potassium current is greater (3, 34). Animal studies suggest a wide safety margin is present for the cardiac sodium current, with a loss of ≥ 50% in association with other factors such as age and sex being necessary to generate a marked phenotype (35). As such, SCN5A may be the only single gene for which mutations are able to decrease inward sodium current sufficiently to cause BrS with high penetrance.

Many of the other genes reported to potentially cause BrS modulate Na_V_1.5 expression, trafficking, or function (4). In addition, GWAS studies have identified common variants in both sodium channel interacting proteins and transcription factors associated with Na_V_1.5 expression that are associated with BrS (5, 6). Thus, the majority of cases of BrS may be polygenic, with decreased inward sodium current related to both rare and common variants in a number of genes, in association with metabolic and environmental factors.

In the multigenerational family with the putative GPD1L mutation studied here, we identified two common variants associated with BrS that were overrepresented compared to the general population. These variants, one in SCN5A and one in SCN10A, are linked to the mutant GPD1L allele in 12 of the 14 affected individuals. The presence of these common variants in association with the mutant GPD1L allele could exacerbate the loss of sodium current, and explain in part why the relatively common A280V-GPD1L variant is pathogenic in the family presented here.

### 4.3 Clinical implications for Brugada Syndrome

Genetic testing is now standard in evaluation of patients with suspected sudden cardiac death syndromes. Physicians and genetic counselors can test for mutations in a single gene, in gene panels, in the exome, or in the genome. While useful, present-day testing results only offer an approximately 25% identification rate in BrS (36). Current recommendations for clinical genetic testing panels in BrS suggest exclusion of all genes except SCN5A. While the intent to avoid undue harm from provider misinterpretation of variants in unproven genes is well-intentioned, this could reduce identification of novel rare variation contributing to BrS in sodium channel modifying genes, among others, and prevent acquiring the data that would prove causality, such as linkage in a large family. We believe these concerns highlight the necessity of appropriate interpretation of genetic testing panels through cardiologists trained in genetics and genetic counselors. We also note the need for widespread reporting of identified variants and research laboratories that can identify and assess putative mutations (37, 38).

### 4.4 Study limitations and future directions

Our study has a number of limitations. First, we used linkage and exome sequencing data from a single family. Linkage data provide an incomplete picture of the genetic architecture for a disease, though we believe the addition of exome sequencing partly addresses this limitation. Notably, exome sequencing libraries are not targeted to capture intronic sequences, and deep intronic variants within *GPD1L* or nearby genes may contribute to disease in this family. Second, we assessed only a fraction of the common SNPs that are associated with BrS (6). Because only SCN5A and SCN10A are near the linkage area on chromosome 3p, we would not expect that the other variants would be heavily overexpressed or underexpressed in our affected individuals. Third, the mechanism by which GPD1L variants alter Na_V_1.5 expression and/or function is not addressed. GPD1L is involved in the metabolic regulation of cellular activity, and this is an area of active investigation using heterologous expression systems and gene-targeted mice.

## 5 Conclusion

We identified GPD1L-A280V as the only rare non-synonymous coding variation in the refined linkage region in a large, multigenerational family. Associated BrS risk SNPs may be permissive for the BrS phenotype in affected family members. In addition, the A280V mutation may alter the GPD1L tertiary structure. While mutation in *SCN5A* is the most common cause genetically-identified cause of BrS, our study suggests that GPD1L should still be considered when evaluating the genetic underpinnings of BrS and sudden death. Ultimately, continued study of BrS through genome-wide analyses, targeted familial sequencing studies, mechanistic studies, and investigation of putative mutations will further our knowledge of this disease.

## Data availability statement

The original contributions presented in the study are publicly available. This data can be found here: https://www.ncbi.nlm.nih.gov/projects/gap/cgi-bin/study.cgi?study_id=phs003468.v1.p1.

## Ethics statement

The studies involving human participants were reviewed and approved by Institutional review boards of the University of Pittsburgh and the University of Iowa. Written informed consent to participate in this study was provided by the participants' legal guardian/next of kin. Written informed consent was obtained from the individual(s), and minor(s)' legal guardian/next of kin, for the publication of any potentially identifiable images or data included in this article.

## Author contributions

AG and BL were responsible for the conception and design of the work. RG and BL enrolled the family, acquired clinical records, and obtained DNA from the family. AG, CC, and HM were responsible for the acquisition and analysis of the work. AG, HM, CC, RG, and BL interpreted data contained in this work. All authors contributed to the process of manuscript drafting and revision.
