# Supplementary material for: The role of GPD1L, a sodium channel interacting gene, in the pathogenesis of Brugada Syndrome
Source: Front Med (Lausanne). 2024 Jun 19;10:1159586. doi: 10.3389/fmed.2023.1159586 (PMC11221213; doi:10.3389/fmed.2023.1159586)

Supplemental Table 1: Illumina Global Sequencing Array Call Rate

**Sample**	**Call Rate**
1	0.9955
2	0.9937
3	0.9955
4	0.9950
5	0.9948
6	0.9953
7	0.9955
8	0.9954
9	0.9955
10	0.9919
11	0.9923
12	0.9865
13	0.9938
14	0.9954
15	0.9955

Supplemental Figure 1: **Whole exome sequencing coverage**. Good coverage was achieved for the Agilent SureSelect V6+UTR kit, with >80% of bases covered above 50x.

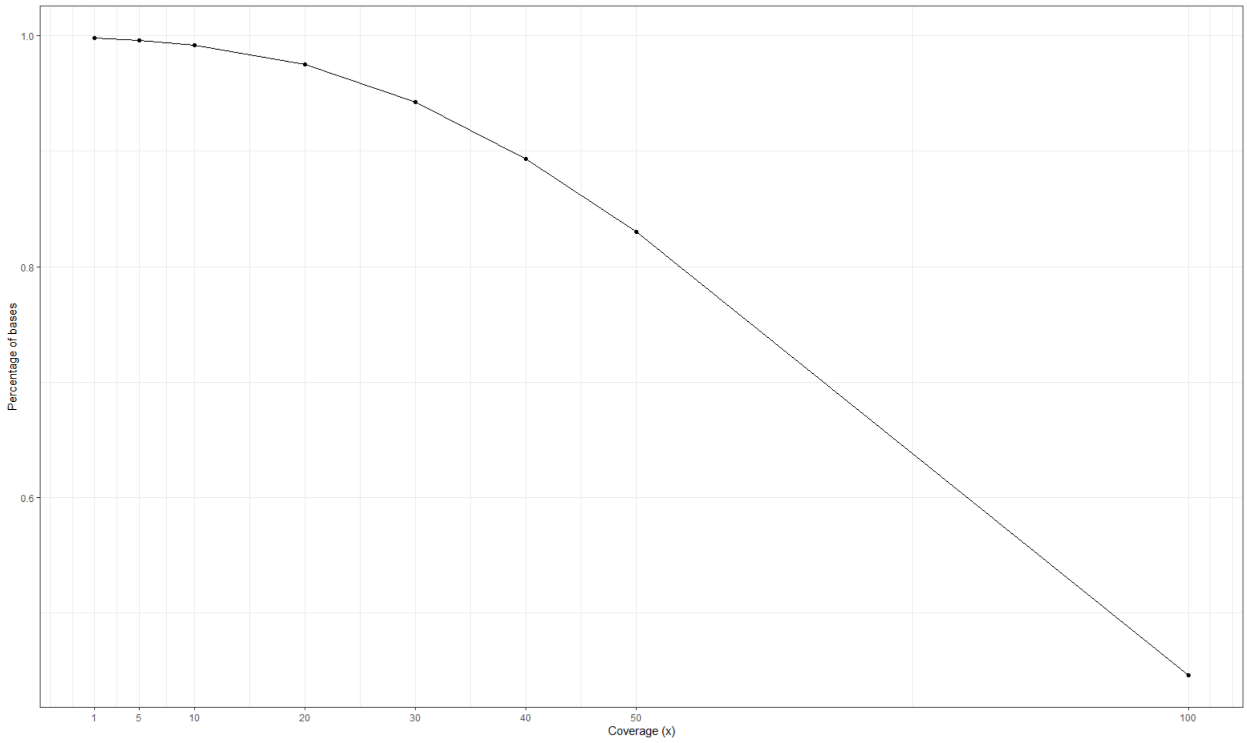

Supplemental Table 2: Whole exome sequencing metrics

**Number of variants**	166834

Variants by type

Type	Count	Percent
DEL	14624	8.77
INS	12256	7.35
SNP	139954	83.89

Variants by effect

Effect	Count	Percent
MISSENSE	34535	47.26
NONSENSE	308	0.42
SILENT	38238	52.32

Supplemental Table 3: Primers used for Sanger Sequencing of Brugada Syndrome Risk SNPs

Primer	Gene	Sequence
rs11708996 F1	SCN5A	TGTTGAGTTGTAGGGTACACAGT
rs11708996 R1	SCN5A	GGGATTGGGAAGGCCTTCAA
rs11708996 F2	SCN5A	ATGTTGATTCCAGTTTCCCCT
rs11708996 R2	SCN5A	TGAACTCACTACCACAAACTGGA
rs10428132 F1	SCN10A	CAGAGCAAATGGAGCAAGGC
rs10428132 R1	SCN10A	CCAGTTCACCAGTCTCCGTC
rs9388451 F1	HEY2	CGAGGTGCCAGGGGTTTTTA
rs9388451 R1	HEY2	GCGAGGAGAATACCAGAGGC
rs9388451 F2	HEY2	CCACAGTGACATGATCCAGGT
rs9388451 R2	HEY2	GGAACATGTGCAAGGCCTTG

Supplemental Figure 2: **GPD1L-A280V PyMol Rotamers**. The AlphaFold predicted model for GPD1L was mutated in PyMol to GPD1L-A280V. Red octagons indicate steric hinderance within GPD1L. A) PyMol predicted rotamer 1 of 3 with a calculated strain of of 14.29. B) PyMol predicated rotamer 2 of 3 with a calculated strain of 14.63. C) PyMol predicted rotamer 3 of 3 20.07

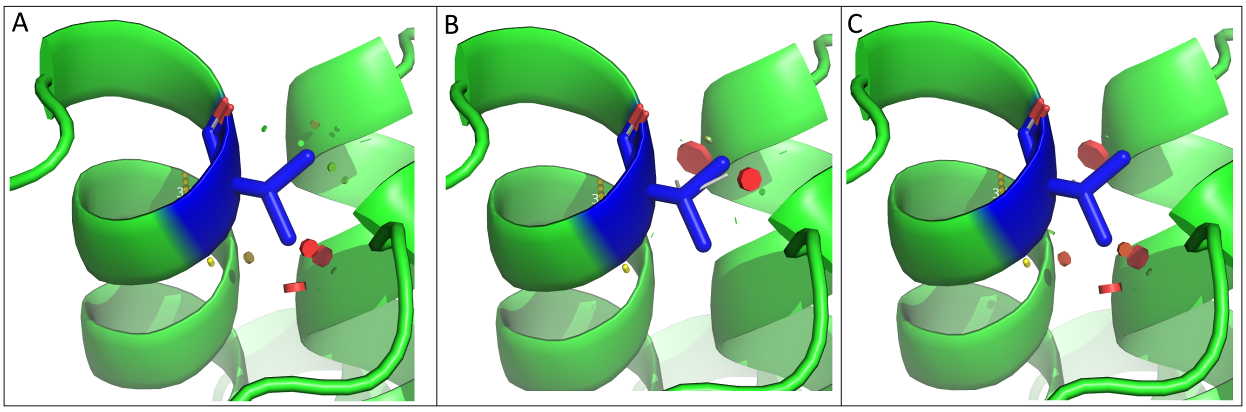

Supplement: Supplementary file 1 [file Data_Sheet_1.PDF]
